# A Cross-Sectional Study about Knowledge, Attitude, and Practices among Primary Health Care Physicians in Jazan Province, Saudi Arabia, Regarding Rome IV Criteria for Diagnosis of Irritable Bowel Syndrome

**DOI:** 10.3390/medicina58121811

**Published:** 2022-12-09

**Authors:** Faisal Abusageah, Alwaleed Hakami, Basem Zogel, Shaden Zaalah, Samar Alfaifi, Sahar Shubayli, Khalid Hakami, Entsar Qadah, Sarah Aldharman, Faisal Hakami, Maram Alqasemi, Mousa Mobarki, Abdulaziz H. Alhazmi

**Affiliations:** 1 Faculty of Medicine, Jazan University, Jazan 45142, Saudi Arabia; 2 Faculty of Medicine, King Saud Bin Abdulaziz University for Health Sciences, Riyadh 14611, Saudi Arabia

**Keywords:** irritable bowel syndrome, primary health care physicians, knowledge, attitude, practice, Jazan, Saudi Arabia

## Abstract

*Background and objectives*: Most individuals with irritable bowel syndrome (IBS) are diagnosed by primary health care (PHC) physicians. However, a significant percentage of patients remain undiagnosed or misdiagnosed owing to the lack of knowledge or a systematic strategy regarding the use of ROME IV criteria for the diagnosis of IBS. Thus, in this study, we aimed to assess the knowledge, attitude, and practices among primary health care physicians in Jazan Province, Saudi Arabia, regarding ROME IV criteria for the diagnosis of IBS. *Methods*: A cross-sectional study was conducted using a pretested self-administered questionnaire that determines participants’ sociodemographic data and measures knowledge about ROME IV criteria, targeting PHC physicians in Jazan Province, Saudi Arabia. Data were analyzed using the Statistical Package for Social Sciences (SPSS) v.23. *Results*: We included 200 participants, and the majority of participants in our study (approximately 78%) were aware of the ROME IV diagnostic criteria for IBS; this awareness was associated with age, nationality, specialty, and classification. The participants’ mean level of knowledge was 4.30 (out of 6). However, knowledge was higher among Saudi and family medicine doctors in this study, as compared to non-Saudi and doctors of other specialties. More than two-thirds of participants who were aware of ROME IV criteria thought that they are sufficient to diagnose IBS; however, only 47.5% of physicians reported using ROME IV frequently in their daily practice. *Conclusions*: Most of the participants of this study are aware of ROME IV criteria, and better knowledge was noted among Saudi and family medicine physicians. About 70% thought that ROME IV criteria are effective enough to diagnose IBS, and only about half of the participants use ROME IV criteria in their practice. Therefore, due to its high prevalence in the region, further efforts are required to disseminate basic knowledge and improve attitudes and practices related to ROME IV criteria among PHC physicians of all specialties.

## 1. Introduction

Irritable bowel syndrome (IBS) is one of the most common functional gastrointestinal disorders and is characterized by recurrent episodes of abdominal pain, change in bowel habits, and bloating, in the absence of any organic and biochemical abnormalities. This disorder has been shown to affect the young age group, with peak incidence in the third and fourth decades of life [1,2]. IBS is a highly prevalent condition with an estimated prevalence ranging from 5% to 10% globally according to a study conducted in 2020 [3]. Regarding the local prevalence in Saudi Arabia, many studies conducted in different regions found that it ranges from 8% to 40% [4,5,6,7,8]. This condition may come with a significant economic burden due to its direct and indirect health care costs [9].

IBS has a negative impact on quality of life, with a strong link to psychiatric disorders, especially anxiety and depression, and those factors could worsen the state of IBS [8,10]. It is a fact that around 33 to 90% of individuals with IBS do not seek medical care, which makes it a major public health problem [2]. The prognosis of IBS is usually benign, but its clinical presentation may mask other serious conditions such as colon cancer [2,11]. The diagnosis of IBS is challenging since there is no specific biochemical or biological marker for it, and symptoms are sometimes difficult to characterize due to the variation in symptoms from one individual to another [11].

The diagnosis is clinically based on certain criteria such as the Manning criteria, Rome I, Rome II, Rome III, and the most recent Rome IV. The Rome IV criteria are frequently utilized in clinical practice and are accepted as the gold standard for IBS diagnosis [12]. These criteria are as follows: recurrent abdominal pain that must have occurred at least twice per week in the previous three months and must be associated with at least two of the following: (i) related to defecation, (ii) change in frequency or (iii) consistency of stool [13]. In addition to the patient’s history, accurate identification and diagnosis of IBS through utilizing the Rome IV criteria is effective and simple to implement by primary health care (PHC) practitioners [14]. It has been recognized that PHC physicians have a significant role in IBS detection and improving the care of the patients it affects [15]. For patients who present without alarming symptoms, PHC practitioners tend to be able to diagnose patients from the first or second visit [4]. However, according to a systematic review study, PHC physicians’ knowledge and application of these criteria are not satisfactory [16], although an appropriate intervention, accurate diagnosis, and optimal care by PHC physicians are crucial for patients with IBS [17,18].

Several studies have been carried out in various countries, including Saudi Arabia; however, there have been very few attempts to assess clinicians’ understanding of the disease and symptoms of IBS in the southwestern region of the country, and there is a dearth of data regarding this topic in Jazan Province. Considering the significant health impact of IBS and the role of PHC physicians in detection and in improving the care of patients, in this study, we aimed to assess the knowledge, attitude, and practices among PHC physicians in our region regarding the ROME IV criteria for the diagnosis of IBS.

## 2. Materials and Methods

### 2.1. Study Design, Setting, and Participants

We conducted this study using a cross-sectional design in Jazan Province, which is located in the southwest of Saudi Arabia. The province is home to approximately 1.7 million people. We included in this study a sample of PHC physicians working in Jazan Province and engaged in practice at the time of the study, and those who refused to participate were excluded.

### 2.2. Sample Size

According to a study conducted in 2020, the total number of PHC physicians in the Jazan region is about 400 [19]. The sample size for this study was calculated using the Raosoft sample size calculator (Raosoft Inc., Seattle, WA, USA, raosoft.com, accessed on 1 June 2022), and 197 were needed to reach a 95% confidence interval and 5% margin of error with 50% as an agreed prevalence.

### 2.3. Sampling Strategy

All the PHC centers in the Jazan region were listed and a random selection was made for particular centers; then, all physicians who were willing to participate from each selected center and who met the inclusion criteria in this study were included. The data were collected by multiple data collectors using a self-administered questionnaire (Table S1). The questionnaire was adapted from one previously published [20]. The first part of the questionnaire started with socio-demographic factors such as age, gender, nationality, marital status, specialty, physician level, years of practice, and monthly income. Then, the second part involved three questions to assess general awareness of the ROME IV criteria, six questions for knowledge, two questions for attitude, and five questions for practice. However, the participants who had not heard of the ROME IV criteria were excluded from answering the remaining questions. Furthermore, the physicians who had not used ROME IV criteria were excluded from answering the practice questions. The ROME IV criteria for the diagnosis of IBS are well established [21]; briefly, the guidelines state the following: This condition must be present over the previous three months, with the onset of symptoms occurring at least six months before the diagnosis. The symptoms are abdominal pain that occurs frequently, at least once daily or once weekly in the past three months, with at least two of the following: related to defecation, associated with altered stool frequency, altered stool form, or altered stool appearance [21].

### 2.4. Pilot Study

Before the distribution of the survey, a pilot study was applied among 15 PHC physicians who were not a part of this study to assess whether the questionnaire items were clear and understandable and to assess the time consumed by filling out the questionnaire.

### 2.5. Data Analysis

Data were analyzed using the Statistical Package for Social Sciences (SPSS version 23, IB, Chicago, IL, USA). For categorical data, frequencies and percentages were used, while for quantitative data, means and standard deviations were used. Chi-square test, *t*-test, and ANOVA were used to identify the socio-demographic factors associated with knowledge and awareness of the ROME IV criteria in the diagnosis of IBS. Regarding knowledge, the score consisted of six items, with each question adding a possible 1 to the score for a total of 6; then, the mean and standard deviation were calculated from the collected scores. A *p*-value of ≤0.05 was considered the cut-off point for statistically significant differences between variables.

### 2.6. Ethical Considerations

Ethical approval to conduct this study was obtained from the Scientific Research Ethics Committee (REC) at Jazan University in Saudi Arabia (reference number: REC-43/11/265; date: 12 June 2022). Informed consent was obtained from all participants. Privacy and confidentiality were preserved, and none of the participants were asked about anything that could reveal their identity or personal information.

## 3. Results

### 3.1. General Characteristics of the Participants

Table 1 provides a detailed description of the participants’ characteristics; the questionnaire was filled out by a total of 200 participants. Most of the participants were male (n = 112; 56%) with a mean age of 36.32 years (SD: 7.981). More than half of the participants were non-Saudi, and a large majority of the participants were married (n = 158; 79%). About 54.5% of the participants were working as general practitioners, while family medicine physicians and physicians from other specialties (general medicine, pediatrics, etc.) accounted for 43% and 2.5%, respectively. A majority of the physicians had been practicing for over five years (61%). Only 4% of the physicians were consultants, and the majority were either general practitioners, residents, or specialists, accounting for 41%, 30.5%, and 24.5%, respectively.

### 3.2. Awareness of ROME IV Criteria as a Diagnostic Tool for IBS

As shown in Table 2, most of the participants in our study (about 78%) were aware of the ROME IV criteria for the diagnosis of IBS, whereas approximately 22% of the participants had not heard of the ROME IV criteria. However, age, nationality, specialty, and classification were significantly associated with better awareness (*p*-value = 0.022, 0.001, 0.001, and 0.002, respectively).

### 3.3. Factors Affecting Levels of Knowledge of ROME IV

Table 3 describes the associations between knowledge of the ROME IV criteria and the socio-demographic factors of the participants. The included participants in this table were those who had heard about ROME IV (n = 155). The mean knowledge score among the participants was 4.30 (*SD* = 1.30). This knowledge was significantly associated with nationality, as it was higher among Saudis compared to non-Saudis, with a *p*-value of 0.003. Furthermore, knowledge was significantly associated with specialty, as a higher score was recorded by family medicine physicians (*M* = 4.59, *SD* = 1.10) compared to others (*p* = 0.001). However, age, gender, marital status, classification, and years of practice did not impact the level of knowledge of the ROME IV criteria.

### 3.4. Attitudes and Practices toward ROME IV among the Participants

Considering the attitude of the physicians toward the ROME IV criteria, Table 4 provides a descriptive analysis of the participants who already used the ROME IV criteria in their daily practice, and it shows that more than two-thirds of the participants considered it effective enough to diagnose IBS. However, 59% of the physicians believed that about 50% of patients qualify for the ROME IV criteria. Table 5 provides data regarding the use of the ROME IV criteria in practice. About 47.5% of the physicians used the ROME IV criteria. Nearly 76% of the participants had used ROME IV at least once during their time practicing as physicians. Almost 44% of the physicians said that they always maintain continuity of care for IBS patients, whereas a majority of the physicians (78%) consider referral to a specialist if the patient shows any alarming features. About 95% of the physicians have participated in raising awareness of the ROME IV criteria.

## 4. Discussion

IBS is highly prevalent in Saudi Arabia, and yet, studies about the knowledge and attitude of physicians toward this disease are limited [4,5,6,7,8,9]. Thus, this study was conducted to evaluate the self-reported knowledge, attitude, and practices among PHC physicians in Jazan Province, southwestern Saudi Arabia, regarding ROME IV criteria for diagnosing IBS. We found in this study that over three-quarters of the participants were familiar with the ROME IV criteria. In addition, family medicine physicians possessed a greater level of knowledge compared to other general practitioners and other specialties.

In Saudi Arabia, one study was conducted in Riyadh and found a higher level of awareness (86%) among PHC physicians regarding the ROME IV criteria compared to that in the current study (78%) (Table 2) [20]. However, the awareness level in this study is still higher compared to that in previous research from Iceland conducted in 2012, which was found to be 65% [22]. Further, nearly half of interns were familiar with IBS diagnostic criteria in one survey conducted in Mysore in 2018 [23], and one-third of Italian family doctors were familiar with ROME IV according to a study conducted in 2005 [24]. Additionally, a systematic review conducted in 2014 demonstrated that only 2–36% of PHC doctors were aware of the ROME IV criteria for IBS [16]. However, the number of years of practice was not associated with better knowledge of ROME IV criteria, and this finding is consistent with the study conducted in Riyadh in 2020 [20]. Taken together, the noted variations in the levels of awareness and knowledge may be attributed to the different rates of prevalence of the disease and the nature of the studied population and training programs, as we (and others) noted significant correlations of age, nationality, specialty, and classification with increased level of awareness (Table 2 and Table 3) [20,25,26]. Thus, we recommend that the training residency programs in the concerned countries should focus on IBS and ROME IV to enrich the knowledge of the trainees, an action that would result in faster diagnosis and better management.

The use of ROME IV as a diagnostic instrument in the diagnosis of IBS, in both primary and secondary care settings, is widely accepted as standard practice [27,28,29]. Thus, the physicians in this study who already used ROME IV are no exception, as more than two-thirds of the participants believed that the ROME IV criteria are adequate for diagnosing IBS (Table 4 and Table 5). However, around three-quarters of the participants had utilized ROME IV at least once, but less than half of the participants used these criteria regularly (Table 5). This finding was slightly different from those of other studies in Riyadh and the northern region of Saudi Arabia, which reported 29.4% and 23% of participants having used ROME IV criteria, respectively [9,20]. In Europe, a study conducted in different European countries in 2017 found that almost one-third (36%) of general practitioners frequently use ROME IV criteria in their daily practice [25]. In this study, a majority of the physicians (59%) believed that only half of patients qualify for ROME IV criteria. This finding is relatively similar to what others observed [20], in which one-third of participants believed that about 50% of the patients qualify for the application of ROME IV criteria. In terms of continuity of care, we discovered that less than half of the included physicians always provide continuity of care for IBS. This might be because this disorder is difficult to manage when considering its psychological and mental consequences [28,29,30], and this finding varied from the findings of other local studies conducted in Riyadh (12.6%) or the northern region of Saudi Arabia (57%) [9,20]. Thus, physicians who deal with patients with IBS must take into account other psychological and cultural perspectives of IBS, in addition to the clinical manifestations that usually force such patients to seek medical consultations. Further, local guidelines for the above-mentioned consequences are deemed essential for better management of this disease.

It is appreciated that the majority of the physicians (95%) in this study had participated in raising awareness of the use of ROME IV criteria (Table 5); this is contrary to a local study that showed that only 43% of physicians participated in raising awareness [20]. It is noticeable in this study that a majority of the physicians acknowledged that ROME IV criteria are effective, and 78% considered a referral to a specialist for patients presenting with alarming features. Likewise, in a study conducted in Lahore, Pakistan, family physicians referred patients with alarming signs to a specialist as those patients should benefit from further evaluation by an expert [31]; this finding is also consistent with a study conducted by Al-Shamrani et al., who found that 65% of the physicians considered a referral to a specialist for patients who developed complications [20]. A possible explanation for referral to a specialist for a second opinion by a majority of the PHC physicians who use ROME IV criteria might be the clarity of the guidelines, which necessitate physicians to recommend patients with alarming signs for an expert opinion to specify diagnostic methods and possible complications [32]. Thus, the benefits of raising awareness of ROME IV are not limited to accurate diagnosis—fewer complications of IBS could be achieved.

This is one of very few studies conducted in Saudi Arabia, a nation that continues to record a high prevalence of IBS, with its non-negligible economic burden, to evaluate the knowledge, attitude, and practices of PHC doctors regarding the ROME IV criteria. It is noteworthy to mention that the findings of this study do not reflect the quality of patient care by any means. Thus, we believe this may evoke the need for enhanced programs to assure better practice and to create, or update, the currently used guidelines in the region. However, this study possesses many limitations. This was a cross-sectional study, so it is expected to have the limitations associated with such a methodology. Another limitation is that the study does not provide a sufficient explanation for the observed variations in the level of knowledge between Saudi and non-Saudi physicians, except for the nature of training programs locally practiced in Saudi Arabia. Further, we did not use a scoring system to evaluate both attitude and practice, like the scoring system we used for knowledge. Finally, the effect of recall and selection bias cannot be ignored as this study was conducted by means of a survey that should be completed via an online platform. Thus, those who did not participate as they had no access to the internet or were less familiar with the platform may affect the overall results.

## 5. Conclusions

In conclusion, our study showed an appropriate level of awareness of ROME IV criteria among PHC physicians for the diagnosis of IBS cases, but their practices towards this condition were inappropriate, as more than half of the participants were not using ROME IV criteria frequently in their daily practice to diagnose IBS. Despite this, 95% of physicians had participated in raising awareness of the use of ROME IV. In this study, the level of knowledge was significantly associated with nationality and specialty, as Saudi physicians and family medicine physicians had better knowledge. Therefore, further efforts are required to increase awareness about the importance of using ROME IV criteria through structured programs to improve the awareness and practice of PHC physicians in Jazan, an action that would result in better diagnosis and less complicated cases of IBS. National studies with a wider scope and a larger sample size are recommended to confirm these findings.

## Figures and Tables

**Table 1:** Demographic characteristics of the participants (n = 200).

Age in Years		
Mean	Median	Standard Deviation
36.32	35	7.981
Variable	n	%
Gender		
Male	112	56%
Female	88	44%
Nationality		
Saudi	87	43.5%
Non-Saudi	113	56.5%
Marital Status		
Married	158	79%
Not Married	42	21%
Speciality		
General Practitioner	109	54.5%
Family Medicine Physician	86	43%
Others	5	2.5%
Classification		
General Practitioner	82	41%
Resident	61	30.5%
Specialist	49	24.5%
Consultant	8	4%
Years of Practice		
Less than Three Years	35	17.5%
From Three to Five Years	43	21.5%
More than Five Years	122	61%

**Table 2:** Awareness of ROME IV criteria among the included participants (n = 200).

Factor		Heard about Rome IV Criteria (n = 155)	Not Heard about Rome IV Criteria (n = 45)	*p*-Value
Age group	(Mean; SD)	36; 7.74	39; 8.4	0.022 *
Gender	Male	89 (57.4%)	23 (51.1%)	0.453
	Female	66 (42.6%)	22 (48.9%)	
Nationality	Saudi	79 (51%)	8 (17.8%)	0.001 *
	Non-Saudi	76 (49%)	37 (82.2%)	
Marital status	Married	121 (78.1%)	37 (82.2%)	0.547
	Not married	34 (21.9%)	8 (17.8%)	
Specialty	General practitioner	71 (45.8%)	38 (84.4%)	0.001 *
	Family medicine	79 (51%)	7 (15.6%)	
	Others	5 (3.2%)	0 (0%)	
Classification	General practitioner	53 (34.2%)	29 (64.4%)	0.002 *
	Resident	50 (32.3%)	11 (24.4%)	
	Specialist	44 (28.4%)	5 (11.1%)	
	Consultant	8 (5.2%)	0 (0%)	
Years of practice	<3 years	31 (20%)	4 (8.9%)	0.174
	3–5 years	34 (21.9%)	9 (20%)	
	>5 years	90 (58.1%)	32 (71.1%)	

SD: Standard deviation. * Significant at *p* < 0.05.

**Table 3:** The association between ROME IV criteria knowledge (depicted as the mean and standard deviation for the calculated score) and socio-demographic characteristics.

Variables		Knowledge about ROME IV Criteria ^#^		*p*-Value
		Mean	Standard Deviation	
Age group ^&^		4.30	1.30	0.774
Gender	Male	4.23	1.28	0.457
	Female	4.39	1.32	
Nationality	Saudi	4.60	1.03	0.003 *
	Non-Saudi	3.98	1.47	
Marital status	Married	4.24	1.33	0.320
	Not Married	4.5	1.29	
Specialty	General practitioner	4.08	1.40	0.001 *
	Family medicine	4.59	1.10	
	Others	2.80	1.30	
Classification	General practitioner	3.96	1.35	0.055
	Resident	4.30	1.46	
	Specialist	4.68	0.98	
	Consultant	4.50	0.92	
Years of practice	<3 years	4.58	1.25	
	3–5 years	4.58	0.98	0.072
	>5 years	4.10	1.39	

SD: standard deviation. ^#^ The score was estimated according to 6 variables measuring ROME IV criteria knowledge (the maximum score was 6, and the minimum was 0). * Significant at *p* < 0.05. ^&^ The mean age of participants was calculated here according to the total number of participants (n = 155) who were eligible for evaluation of their knowledge of the ROME IV criteria.

**Table 4:** Attitude toward ROME IV criteria among PHC physicians.

Variable	Response	%
What proportion of patients qualifies for the ROME IV criteria to be applied for diagnosing IBS?	<25%	14
	25–50%	45
	>50	37
	I don’t know	9
Do you feel that ROME IV criteria are effective enough to diagnose IBS?	Yes	70
	No	12
	I don’t know	17

PHC: primary health care. IBS: irritable bowel syndrome.

**Table 5:** Practice of ROME IV criteria among primary care physicians for the diagnosis of IBS.

Variable	Response	n	%
Have you ever used ROME IV criteria to diagnose IBS?	Yes	118	76%
	No	37	24%
Do you frequently use ROME IV criteria to diagnose IBS?	Yes, for all cases	56	48%
	For selected cases	61	52%
	Not at all	1	1%
Which cases of IBS do you consider for specialist referral? (more than one answer is possible)	Long-duration patients	21	16%
	All patients	4	3%
	Alarming features such as anemia, rectal bleeding, and weight loss	104	78%
	None	3	2%
Are you able to achieve continuity of care for IBS patients?	Always	52	44%
	Sometimes	60	51%
	Rarely	5	4%
	Never	1	1%
Have you participated in raising awareness?	Yes	112	95%
	No	6	5%

IBS: irritable bowel syndrome.

## Data Availability

The data presented in this study are available on request from the corresponding author.

## Supplementary Materials

The following supporting information can be downloaded at: https://www.mdpi.com/article/10.3390/medicina58121811/s1, Table S1: Questionnaire used in this study to measure knowledge of ROME IV criteria to diagnose IBS.
