# Supplementary material for: A Cross-Sectional Study about Knowledge, Attitude, and Practices among Primary Health Care Physicians in Jazan Province, Saudi Arabia, Regarding Rome IV Criteria for Diagnosis of Irritable Bowel Syndrome
Source: Medicina (Kaunas). 2022 Dec 9;58(12):1811. doi: 10.3390/medicina58121811 (PMC9785885; doi:10.3390/medicina58121811)
Supplement: Supplementary file 1 [file medicina-58-01811-s001.zip › medicina-2036136-supplementary.pdf]

Supplementary Materials: Table S1: Questionnaire used in this study to measure knowledge of ROME IV criteria to diagnose IBS.

	Question	Answer options
**First**	**Socio-demographic factors:**	
	Age group	20-30 years<br>31-40 years<br>>40 years
	Gender	Male<br>Female
	Nationality	Saudi<br>Non-Saudi
	Marital status	Never been married<br>Been married
	Specialty	Family Medicine<br>Others
	Classification	Consultant<br>Specialist<br>Resident
	Years of practice	<3 years<br>3-5 years<br>>5 years
**Second**	**General Awareness**	
	Have you Heard about Rome IV criteria?	Yes<br>No

**<u>Third</u>**	**<u>Knowledge about the components of ROME IV criteria:</u>**	
	Symptoms of IBS or functional bowel disorder that are addressed in ROME IV criteria (*<u>multiple answers can be chosen</u>*)	Recurrent abdominal pain<br>Relation of abdominal pain to defecation<br>Associated with change in stool frequency<br>Associated with change in stool appearance
	The symptom onset should be for how much duration to apply ROME criteria.	>6 months (correct response)<br><6 months<br>No relation<br>I don't know
	The criteria should be fulfilled for how much of the time duration.	1 month<br>2 months<br>3 months (correct response)<br>6 months<br>I don't know
**<u>Fourth:</u>**	**<u>Attitude towards ROME IV criteria for diagnosing IBS:</u>**	
	In your opinion what proportion of patients qualify for the ROME criteria to be applied for diagnosing IBS?	<25%<br>25 – 50%<br>>50<br>I don't know.
	Do you feel that ROME criteria are effective enough to diagnose IBS?	Yes<br>No<br>I don't know.
**<u>Fifth:</u>**	**<u>Practice Component</u>**	
	Have you ever used ROME IV criteria to diagnose IBS?	Yes<br>No
	Do you frequently use ROME IV criteria to diagnose IBS?	Yes, for all cases

		For selected cases<br>Don’t use it at all
	Which cases of IBS do you consider for specialist referral?	Long duration of patients<br>All patients<br>None<br>Development of complications
	Are you able to achieve continuity of care for IBS patients?	Always<br>Sometimes<br>Rarely<br>Never
	Participated to raise awareness	Yes<br>No
